# Examining the needs, outcomes, and current treatment pathways of 2461 people with Treatment-Resistant Depression: A mixed-methods study

**DOI:** 10.1192/j.eurpsy.2025.680

**Published:** 2025-08-26

**Authors:** K. Gill, D. Hett, M. Carlish, R. Amos, A. Khatibi, I. Morales-Munoz, S. Marwaha

**Affiliations:** 1 School of Psychology, Institute for Mental Health; 2 Birmingham and Solihull Mental Health NHS Foundation Trust, National Centre for Mental Health, Birmingham, United Kingdom

## Abstract

**Introduction:**

Major Depressive Disorder (MDD) is a pervasive global health issue, contributing significantly to disability and impaired quality of life. A substantial proportion of individuals with MDD develop Treatment-Resistant Depression (TRD), characterised by the failure to respond to at least two adequate antidepressant treatments (at an adequate dose and duration). TRD poses additional challenges due to its complex clinical presentation and limited treatment options, making it crucial to better understand its impact and develop more effective care strategies.

**Objectives:**

To investigate the prevalence and clinical profiles of TRD in a large NHS Mental Health Trust and explore the treatment experiences and perceptions of TRD patients and healthcare professionals (HCPs) involved in their care.

**Methods:**

A concurrent mixed-methods approach, incorporating patient and public involvement (PPIE), was used. Quantitative analysis of anonymised electronic health records (EHRs) identified the TRD cohort and key characteristics (e.g. age, gender, employment status). Binary logistic regression explored predictors such as comorbidities and service use. The qualitative component included semi-structured interviews with TRD patients (n=7) and HCPs (n=8), analysed using thematic analysis to explore lived experiences and treatment barriers. Findings from both approaches were integrated to provide a comprehensive understanding of TRD.

**Results:**

TRD was prevalent in 48% of patients diagnosed with MDD. Predictors of TRD included recurrent depression (OR=1.24, CI 95%=1.05–1.45), comorbid anxiety (OR=1.21, CI 95%=1.03–1.41), personality disorders (OR=1.35, CI 95%=1.10–1.65), and cardiovascular diseases (OR=1.46, CI 95%=1.02–2.07). Qualitative findings highlighted the severe emotional impact of TRD on patients’ lives and revealed significant dissatisfaction with treatment options, particularly frustration with the “trial and error” approach of pharmacological treatments. HCPs echoed concerns about the lack of standardised treatment pathways, with both groups emphasising the need for more holistic and personalised care, citing limited access as a serious barrier to effective treatment.

**Conclusions:**

This study highlights the significant burden of TRD, affecting nearly half of MDD patients within the examined NHS Trust. By combining quantitative and qualitative methods, it offers a comprehensive understanding of TRD’s prevalence and complexities. The findings support a shift toward holistic, patient-centred care, addressing institutional barriers and enhancing healthcare provider resources to improve outcomes.

**Disclosure of Interest:**

None Declared

